# BUILDING BRIDGES ACROSS AGE-FRIENDLY ELEMENTS GUIDED BY THE 4MS FRAMEWORK

**DOI:** 10.1093/geroni/igad104.1364

**Published:** 2023-12-21

**Authors:** Darina Petrovsky, Anne Mitchell

## Abstract

The 4 Ms framework is a set of evidence-based elements of high-quality care delivered to older adults. These include What Matters, Mobility, Medication, and Mentation. By following these principles and practicing age-friendly older adults are more likely to experience good quality care that addresses their needs. This symposium aims to build bridges across age-friendly elements that focus on the 4 Ms framework by highlighting the research of four nurse scientists. Representing the “What Matters” element, advance care planning and palliative care interventions aim at understanding and aligning care with older adults’ care preferences. In the first presentation, Dr. Rahemi will present findings of health disparities across sociodemographic groups in advance care planning using data from the Health and Retirement Study. In the second presentation, Dr. Carpenter will report on findings from a pilot pragmatic clinical trial that examined the feasibility, acceptability, and preliminary effectiveness of a primary palliative care intervention among nursing home residents with serious illness. Under the element of “Mobility”, Dr. Sefcik will present findings from a qualitative descriptive study aimed at discovering how the COVID-19 pandemic affected the outdoor activities of senior living community-residing older adults. In the fourth presentation, Ms. Coates presentation examines the last two elements of the framework - Medication and Mentation. She will report on findings from a longitudinal case-control observational study using data from the National Health and Aging Trends Study examining differences in the frequency of unpleasant symptoms experienced by older adults with polypharmacy and/or dementia.

